# Supplementary figures and images for: Body weight, gonadectomy, and other risk factors for diagnosis of osteoarthritis in companion dogs
Source: Front Vet Sci. 2023 Nov 28;10:1275964. doi: 10.3389/fvets.2023.1275964 (PMC10713818; doi:10.3389/fvets.2023.1275964)

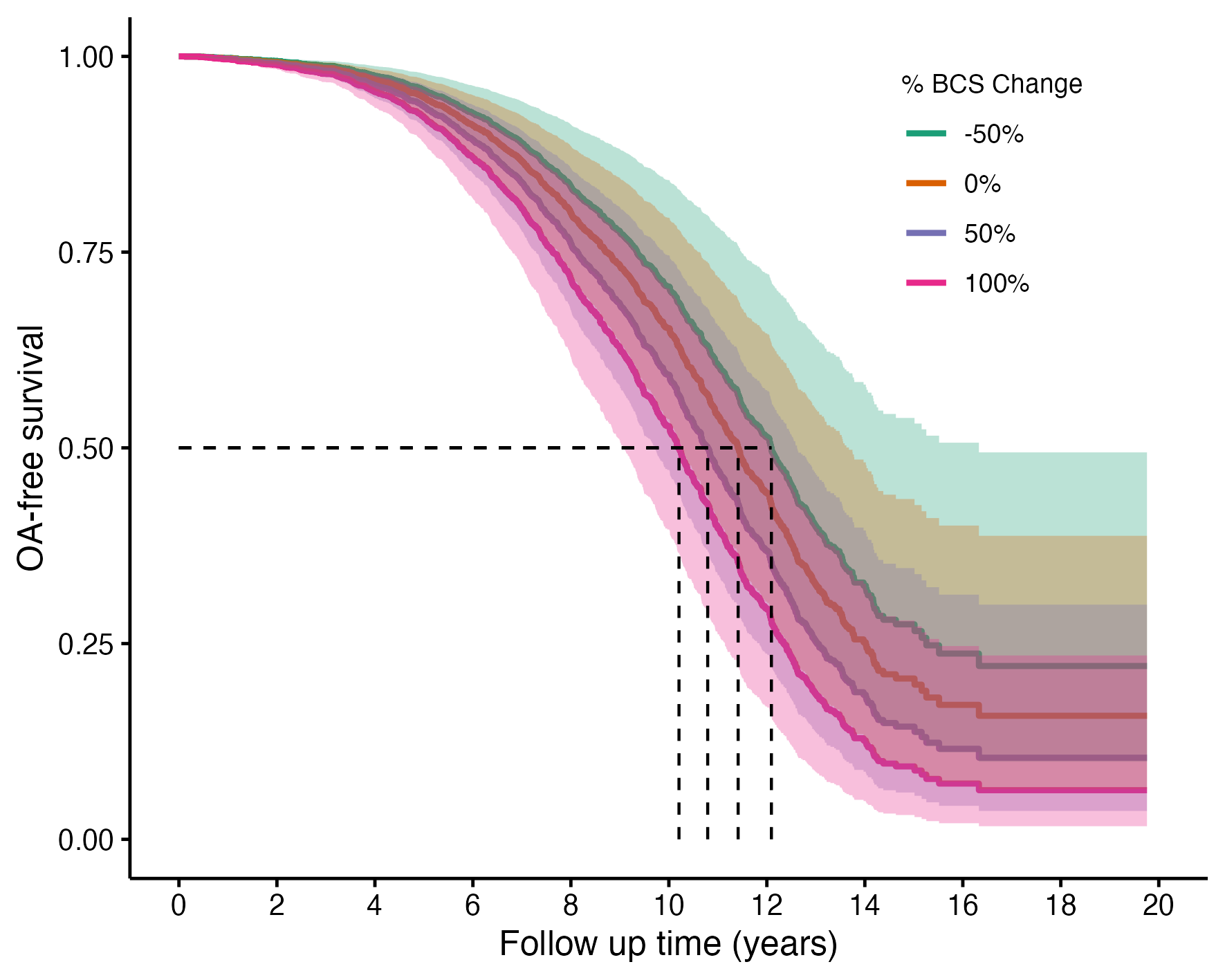

Supplement: Supplementary file 2 [file Image_5.TIFF]

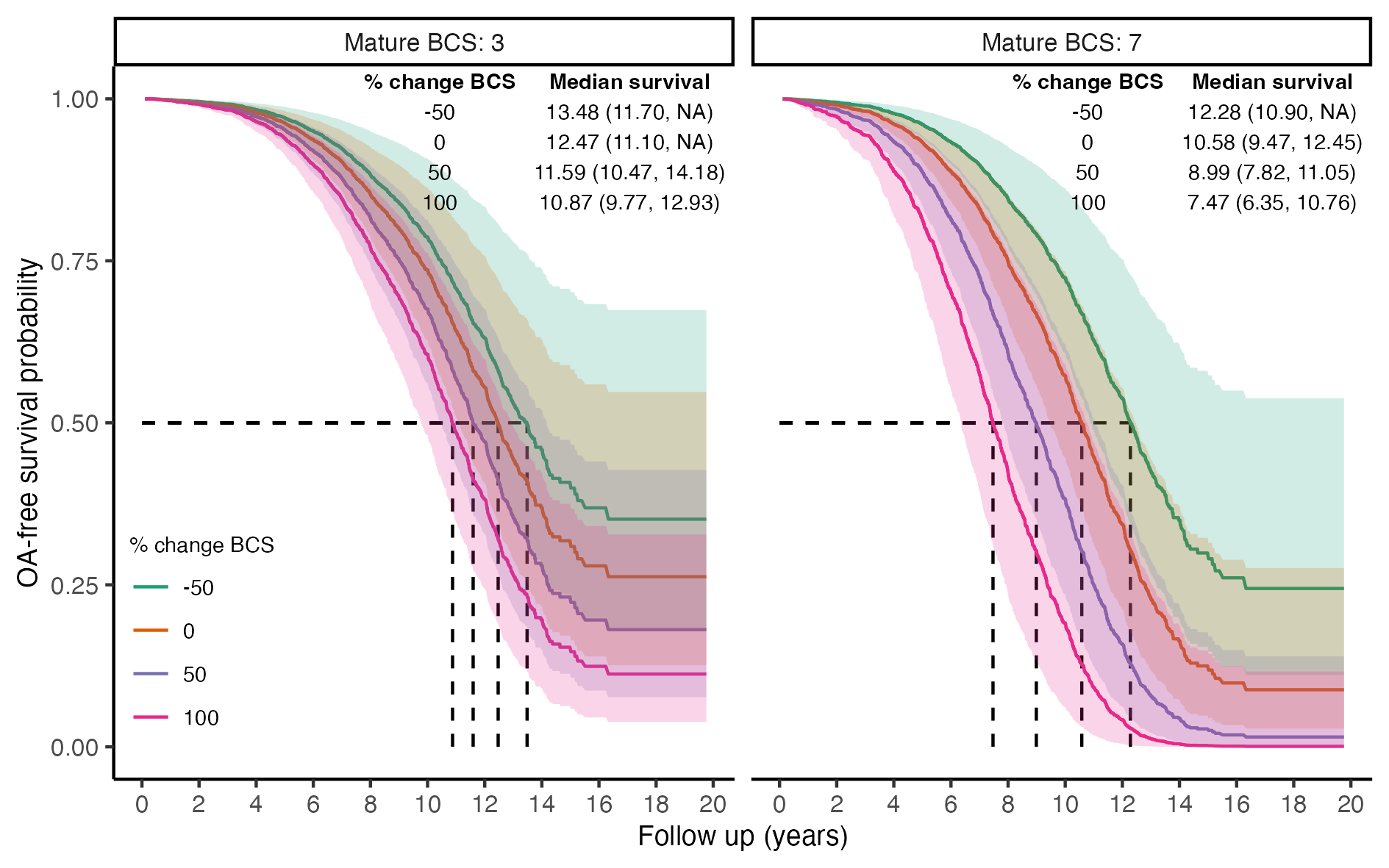

Supplement: Supplementary file 3 [file Image_6.TIFF]
